# Structural assembly of two-domain proteins by rigid-body docking

**DOI:** 10.1186/1471-2105-9-441

**Published:** 2008-10-16

**Authors:** Tammy MK Cheng, Tom L Blundell, Juan Fernandez-Recio

**Affiliations:** 1 Department of Biochemistry, University of Cambridge, 80 Tennis Court Road, Cambridge CB2 1GA, UK; 2 Barcelona Supercomputing Center, Calle Jordi Girona 29, E-08034 Barcelona, Spain

## Abstract

**Background:**

Modelling proteins with multiple domains is one of the central challenges in Structural Biology. Although homology modelling has successfully been applied for prediction of protein structures, very often domain-domain interactions cannot be inferred from the structures of homologues and their prediction requires *ab initio *methods. Here we present a new structural prediction approach for modelling two-domain proteins based on rigid-body domain-domain docking.

**Results:**

Here we focus on interacting domain pairs that are part of the same peptide chain and thus have an inter-domain peptide region (so called linker). We have developed a method called pyDockTET (**tet**hered-docking), which uses rigid-body docking to generate domain-domain poses that are further scored by binding energy and a pseudo-energy term based on restraints derived from linker end-to-end distances. The method has been benchmarked on a set of 77 non-redundant pairs of domains with available X-ray structure. We have evaluated the docking method ZDOCK, which is able to generate acceptable domain-domain orientations in 51 out of the 77 cases. Among them, our method pyDockTET finds the correct assembly within the top 10 solutions in over 60% of the cases. As a further test, on a subset of 20 pairs where domains were built by homology modelling, ZDOCK generates acceptable orientations in 13 out of the 20 cases, among which the correct assembly is ranked lower than 10 in around 70% of the cases by our pyDockTET method.

**Conclusion:**

Our results show that rigid-body docking approach plus energy scoring and linker-based restraints are useful for modelling domain-domain interactions. These positive results will encourage development of new methods for structural prediction of macromolecules with multiple (more than two) domains.

## Background

It is estimated that two thirds of proteins in prokaryotes and four fifths of those in eukaryotes are multi-domain proteins [1,2], many of which have important functions in cell regulation and signalling. From a structural point of view, they range from those with significant and stable interactions between domains, which can usually be defined by X-ray and NMR, to those with flexible linkers and few domain-domain interactions that endow them with large conformational freedom. Crystallography of multi-domain proteins that have flexible linkers is more problematic. X-ray crystallography and NMR approaches have tended to adopt a "divide-and-conquer" approach, by first defining structures of individual domains, although their structures have often been determined within multi-protein complexes where the relationships between domains are often well defined.

For multi-domain proteins with no structural information, their domain orientations may be predicted through homology modelling. However, homologous multi-domain templates are not always available. Furthermore, even if a homologous template exists, its domains might not interact in the same way as the protein to model (see the review of Aloy and Russell [3]). To minimize the chance of inferring wrong interaction data from the templates, Aloy and Russell tried to model putative interactions by assessing residue contacts in the interfaces of known three-dimensional protein structures [4]. Thus, in addition to homology modelling, there has been increasing focus on *ab initio *approaches. For instance, Wollacott and co-workers [5] modelled domain-domain assemblies by placing the domains at the N- and C-terminal of the linker structure, whose conformation is sampled during the procedure Their approach successfully identified near-native assemblies in 50% of the studied cases.

Another promising tool for *ab initio *modelling of multi-domain proteins is docking. Rigid-body docking approaches have already shown success in predicting interactions between relatively rigid globular protomers in protein complexes, as seen in the recent CAPRI (Critical Assessment of PRedicted Interactions; ) blind tests. However, although protein-protein docking could be directly applied to model domain-domain interactions, only a few specific cases have been reported (perhaps because ranking of domain-domain poses is still challenging). As an example, vitronectin was reportedly modelled by docking two of its domains [6], but it required a strong inter-domain constraint from a disulfide cross-link. Lise and co-workers have developed an approach for docking two domains that are part of the same protein chain [7], using pair-wise residue contact function, which includes structural, physicochemical and evolutionary information, to distinguish the native-like domain assemblies from other solutions generated by standard docking procedures. Their work suggests that data-driven docking is useful in modelling domain assembly as well. Furthermore, Inbar and co-workers [8] have extended the docking approach to multi-domain and multi-molecular assemblies, by using a heuristic that applies hierarchical construction to represent the assembly process and a greedy algorithm to select candidate complexes. The modelling of multi-domain proteins has also further promising applications in the field of modelling protein-protein complexes where any of the components has multiple domains. Instead of docking multi-domains directly, the problem can be tackled through divide-and-conquered approaches, which solve the structure of a multi-domain complex by first modelling the orientation of domains within a protein if there are stable relationships, and secondly each domain assembly can then be treated as a protomer for further docking. In this line, Ben-Zeev et al. [9] applied docking between domains with residue conservation restraints to one of the CAPRI targets (T09), as part of a multi-docking protocol, although with limited success (acceptable model ranked 75).

In this paper, we describe a new approach, pyDockTET, for pair-wise assembly of domains that are connected by an inter-domain linker. In addition to the electrostatics and desolvation energy in the original pyDock scoring function [10], which gave one of the best performances for protein-protein docking in the recent CAPRI test [11], an additional pseudo-energy term derived from the end-to-end distance of linkers is incorporated in pyDockTET to select the near-native pair-wise domain poses. We also discuss here the dependence of this scoring function on the linker length and on the quality of the domain models used for the docking.

## Results and discussion

### Structural analysis of linkers in multi-domain proteins

**Figure 1:**
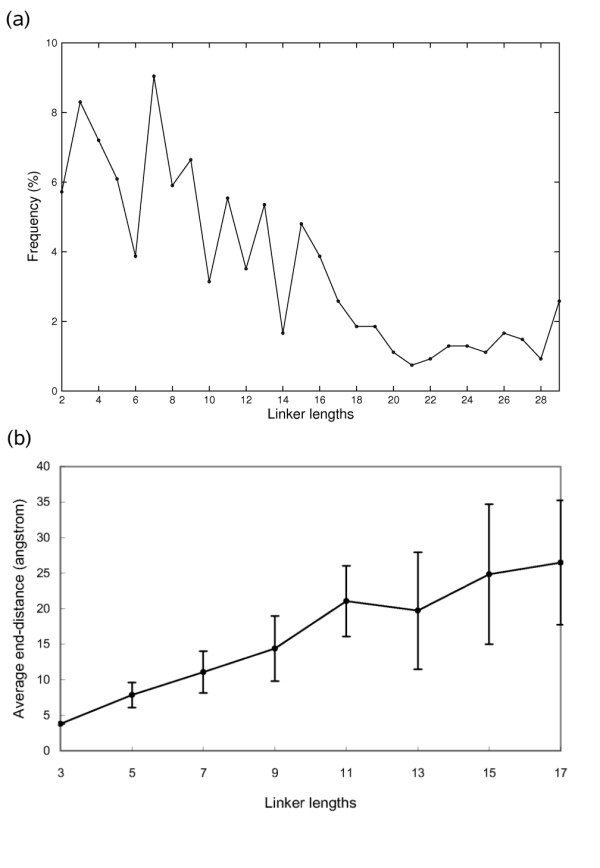
**The relation between length and frequency or end-to-end distance of linkers**. (a) The relation between length and frequency of linkers in our database of 542 linker structures. (b) The relation between length and end-to-end distance of linkers. The length N on the x-axis refers to those linkers that have length with residue number N and N-1. The error bars in the graph indicate the standard deviation for the average end-to-end distance of the length N linkers.

We begin our analysis by examining the inter-domain peptide region (so called linker), whose information is applied in the scoring function of pyDockTET. The goal is to study the relationship between the linker sequence length (in number of residues) and the distance between the linker ends (defined as the distance between the Cα atoms of the two ends of a linker). We began by compiling inter-domain linkers from multi-domain structures in PDB (Protein Data Bank), with the following requisites: i) The linkers were identified as those polypeptide parts placed between domains defined by Pfam [12] (although Pfam may not always defines domains with clear structural boundaries as those defined in SCOP [13], it is more realistic for identifying domains from protein sequences that have unsolved structures); ii) Only structures with a resolution ≤ 2.5Å were used, in order to obtain atomic positions of linkers with greater certainty (we did not either consider NMR structures for this reason); and iii) Only linkers joining domains that are in contact (i.e. there is at least one domain atom at ≤ 5Å distance from any other atom of the other domain) were included. Although prediction of two-domain proteins with no contacts between domains is an important modelling problem, it is not the object of our method (through docking we aim to predict the binding mode of two interacting domains). The sequence length of the 542 linkers in this database varies from 2 to 29 amino acids. The linker distribution shows a higher frequency for linkers with shorter lengths, while linkers of 18 residues or more show lower frequencies (Figure 1a).

**Figure 2:**
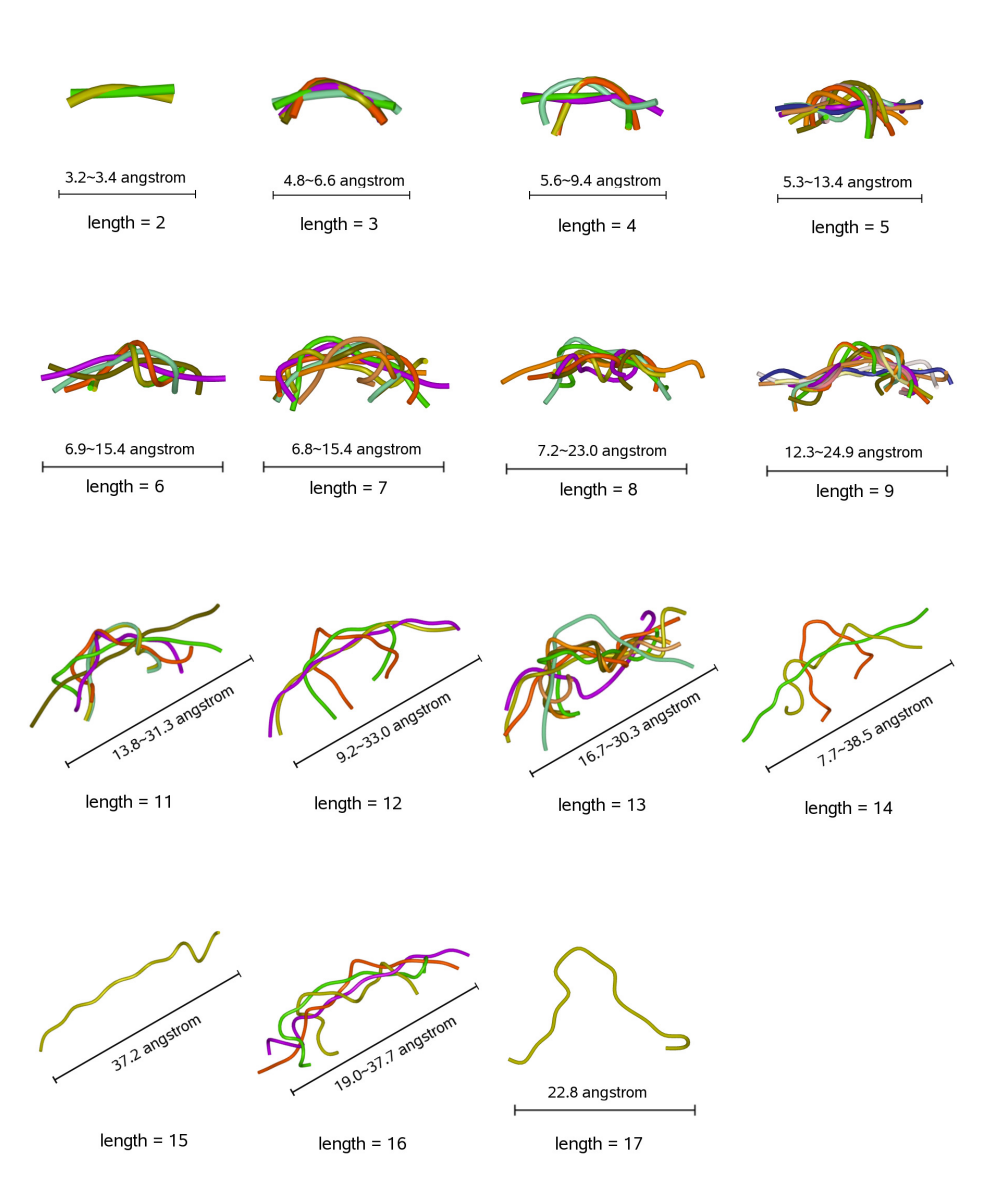
**Inter-domain linkers in our data set**. Inter-domain linkers in our data set classified according to their sequence lengths.

The average linker end-to-end distances increased almost linearly with sequence length for linkers less than 18 residues long (Figure 1b). This dependence is similar to the one described for a statistical ensemble of polymer conformations, where the average end-to-end distance obeys a "random walk Gaussian chain" distribution that scales with L*N^0.5 ^(being *N *the number of residues in linker, and *L *the length of each residue) [14]. This is consistent with the fact that we cannot find conformational preferences in the structural data set for linkers of up to 17 residues length (Figure 2), and with previous studies showing that most linkers lack secondary structure [15]. However, given our broad definition of contacting domain-domain pairs (i.e. at least one inter-domain atomic contact), concern existed that those cases in our data set with very few inter-domain contacts could introduce errors in the derived statistical parameters. In order to disregard this possibility, we removed the 80 cases that had equal or less than 10 contact residues, finding that the statistical parameters (average linker distances and corresponding standard deviations) did not significantly change (data not shown). We also checked that when removing the additional 42 cases that had up to 20 contact residues, the statistical parameters were basically the same (data not shown).

From these average end-to-end distance values and their standard deviations, we have derived a scoring function pyDockTET for docking of domain pairs (see Methods). Given the low frequency (and correspondingly higher variation of average end-to-end distance value) of linkers with length larger than 17, the docking sets we used to benchmark pyDockTET included only domain pairs that have inter-domain linkers with length between 2 – 17 residues.

### Rigid-body docking for domain-domain assembly

**Figure 3:**
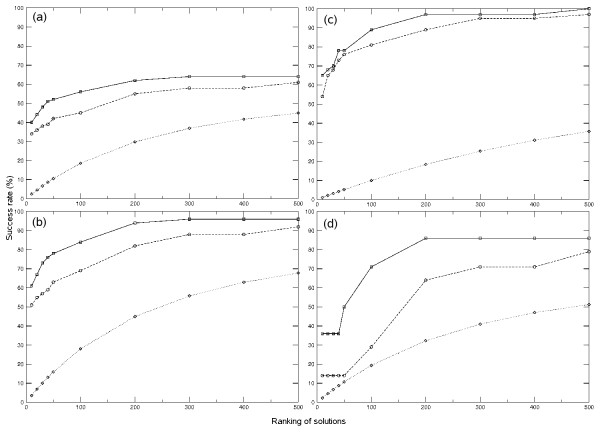
**The overall success rate of pyDock and pyDockTET**. (a) The success rate of pyDock and pyDockTET for identifying acceptable solutions (RMSD = 10 Å) in the top N solutions (N = 10, 20, 30, 40, 50, 100, 200, 300, 400, 500) for the non-redundant domain-domain set. (b) Success rates for identifying acceptable solutions when considering only those cases with at least one acceptable solution within the ZDOCK docking set. (c) Success rates for identifying good solutions (RMSD ≤ 5 Å), considering only those cases with at least one good solution within the ZDOCK docking set. (d) Success rates for identifying acceptable solutions considering only those cases that had acceptable but not good solutions. In the four panels, the results of pyDockTET are shown with solid lines and the results of pyDock are shown with dashed lines with circle markers. The random predictive rates are shown with dotted lines with diamond marker.

We have used docking to rebuild a data set of 77 non-redundant proteins formed by two interacting domains (see Methods). First, we extracted the coordinates of the domains from their X-ray structures, then we separated them and modified all side-chains of the isolated domains with SCWRL 3.0 [16] in order to minimize bias from the use of the assembled structures; finally we docked them as rigid-bodies with ZDOCK [17], and scored the docking poses with pyDock. Docking poses were further rescored with distance restraints derived from the linker database, according to linker sequence length (pyDockTET method). In order to evaluate the success of our predictions, we have defined an acceptable docking pose as one where the RMSD of one of the domains is ≤ 10Å from the equivalent one in the X-ray structure when the other domain (typically the larger one) is superimposed onto that of the X-ray structure (the choice of 10Å RMSD to define an acceptable pose is in concordance with the ligand RMSD used by CAPRI to define an acceptable solution in protein-protein docking). The docking results for all cases, as scored by pyDock and by pyDockTET, are shown in Figure 3a. However, since our goal was to evaluate the success of our scoring function in the identification of the correct domain-domain assemblies within a docking set, we then considered only those cases that had at least one acceptable docking solution within the 2,000 docking poses generated by ZDOCK (51 out of the 77 cases; this is a similar ratio to the one we recently reported for ZDOCK in a standard protein-protein benchmark [10] so it does not seem related to any particular feature of domain-domain interaction). Thus, the use of pyDock method to rebuild inter-domain interactions gave an acceptable pose in the top 10 and top 50 ranked solutions in 51% and 63% of these cases, respectively (Figure 3b), well over expected by random, and comparable to previous rigid-body tests. When we re-scored the docking poses using linker-length distance restraints with pyDockTET, the success rates for top 10 and top 50 solutions increased to 61% and 78%, respectively (Figure 3b). Although the cutoff of 10Å RMSD for defining near-native solutions is accepted by the docking community for assessment of predictions as in CAPRI, the resolution of the resulting models might be too low for some functional predictions. Therefore, we have also evaluated the success rates for detecting a good solution (defined as one with RMSD ≤ 5Å from the X-ray structure). If we consider only those cases that have at least one good solution within the 2,000 docking poses generated by ZDOCK (37 out of the 77 cases), the pyDockTET results (Figure 3c) are comparably good (65% and 78% success rates in the top 10 and top 50 solutions, respectively). In those cases, pyDock alone already had a good performance (54% and 76% success rates in the top 10 and top 50 solutions, respectively), reflecting that when the docking method is able to generate good quality solutions, the pyDock scoring function is already quite accurate. Actually, the restraint-based energy helps especially in those cases where the quality of the docking solutions is not so good (as in the 14 cases that have acceptable but not good solutions; Figure 3d).

The use of pyDock to identify domain assemblies from docking sets clearly gives well over random scoring, and the introduction of linker-based restraints as in pyDockTET further improves the results. Of course, for a realistic case, one has to rely on the docking procedure to generate near-native orientations. We have used here the known FFT-based docking method ZDOCK, but it is expected that the increasing success of rigid-body docking methods will also improve the predictive rates of pyDockTET.

### Dependence of the predictive success of pyDockTET scoring function on linker length

The scoring function of pyDockTET uses the average end-to-end distance for every linker length *L *(*L *= 2, 3, …,17) as a restraint. It is expected that, as the linker length increases, it will provide a less useful restraint on the selection of docking poses, and therefore the performance of the method will likely depend on linker length. Here we analyse the success rates of pyDockTET for different linker lengths, considering only those cases of our domain-domain set that have at least one acceptable solution.

**Figure 4:**
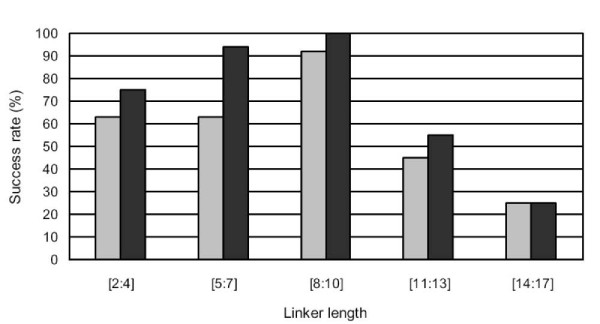
**Comparison of pyDock and pyDockTET according to the linker length between domains**. The success rates of pyDock (light grey bars) and pyDockTET (dark grey bars) in selecting at least one acceptable solution within the top 50 solutions according to the linker length (considering only cases with acceptable solutions).

We classified linker lengths into five groups: 2–4, 5–7, 8–10, 11–13 and 14–17 amino acids. As shown in Figure 4, pyDockTET gave consistently better predictive success rates in top 50 solutions than pyDock, being the improvement of the restraint-based scoring function particularly significant for linker lengths 5–7 (which is also the most populated group). In addition, the overall predictions, both for pyDock and for pyDockTET, increase with linker size up to linker lengths 8–10, and then, for longer linkers, the performance significantly decreases (although for linker lengths 14–17, the number of cases n = 4 is too low for a reliable statistics). According to this, the best scenario for domain-domain assembly by docking and end-to-end restraints would be those cases with linker length up to 10 residues.

### Dependence of the predictive success of pyDockTET scoring function on the type of domain-domain interface: docking energy and number of contacts

The scoring function of pyDockTET consists of a pseudo-energy term derived from linker end-to-end distances, in addition to the original pyDock function that is formed by electrostatics and desolvation energies. We have already shown that pyDockTET function performs in general better than that of pyDock, so now we will analyze in which cases this improvement is more apparent.

**Figure 5:**
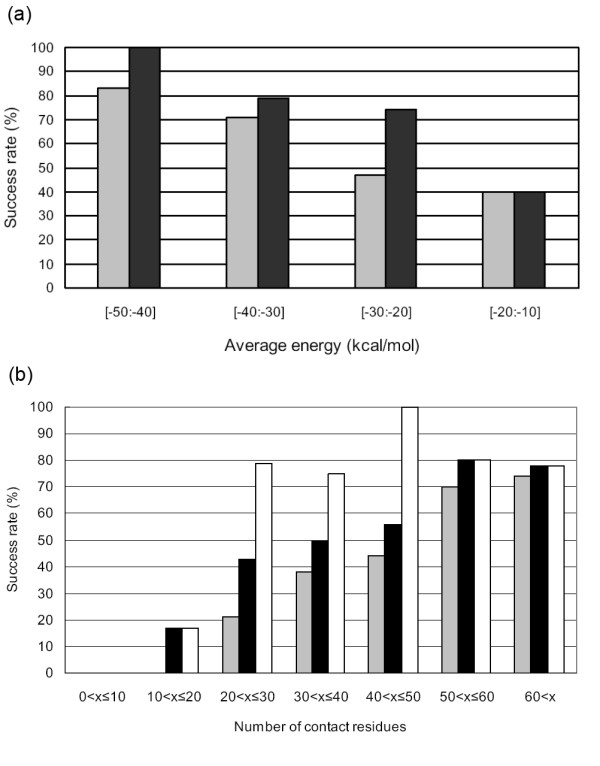
**Comparison of pyDock and pyDockTET according to the energy and the number of contact residues**. (a) The success rates of pyDock (light grey bars) and pyDockTET (dark grey bars) in selecting at least one acceptable solution within the top 50 solutions according to the average electrostatics plus desolvation energy. (b) The success rates of pyDock (light grey bars) and pyDockTET (dark grey bars) according to the number of domain-domain contact residues; the percentage of cases in which ZDOCK generated at least one acceptable solution is also shown (white bars).

First, we will check whether the pyDockTET improvement over pyDock depends on the average pyDock energy obtained for the pool of docking poses, which is different for each case. For each one of the cases that have at least an acceptable docking solution, we sorted the docking solutions by pyDock energy (defined as only electrostatics and desolvation) and computed the average of the best 100 energy values. Figure 5a shows the dependence of the success rates of pyDockTET and pyDock on the average energy (electrostatics plus desolvation) of the top 100 solutions. The overall success rates of both pyDock and pyDockTET increase as the average energy value is lower. Moreover, pyDockTET improves pyDock performance in all cases except for those with average energy value between -20 and -10 kcal/mol (where the low number of cases, n = 5, may produce unreliable statistics), being the improvement particularly good for those cases where pyDock alone gave worse performance (average energy between -30 and -20 kcal/mol). Thus, as would be expected, the most useful contribution made by tethered domain docking is when the average energy value of the top 100 solutions is low.

As for the size of the domain-domain interface, we showed above (the first section of Results) that the existence of cases with low number of inter-domain contact residues did not affect the average linker distances and corresponding standard deviations derived from our data set. However, we observed in Figure 5b that the docking results actually depended quite significantly on the interface size. Figure 5b shows the global success rates of pyDock and pyDockTET, with regard to the number of contact residues in the interface (defined as residues within 5Å distance from any atom of the other domain). It also shows the percentage of cases with acceptable solutions within the docking set (this actually limited the maximum success rates we could expect from pyDock or pyDockTET). Strikingly, ZDOCK found acceptable solutions only in one of the 13 cases with less than 20 contact residues (and no acceptable solution was found for the cases with less than 10 contact residues), which indicates a clear limitation of the FFT-based docking generation. This is in line with previous reports relating docking difficulty and interface size [18,19]. For cases with acceptable docking poses, the pyDock scoring function also showed worse results when the number of contact residues was small – for the cases with less than 30 contact residues, pyDock has success rate at 25% whereas pyDockTET provides a significantly better success rate at 58%. In summary, the linker-based restraints of pyDockTET were able to largely improve the predictive results on those cases particularly difficult for unrestricted docking (i.e. with poor docking energies and/or small number of contact residues).

### Assembly of domains from modelled individual structures

So far we have shown that pyDockTET gives excellent performance in assembling domain pairs where the structures of the two domains have been obtained from crystal structures, after separating them and remodelling the side-chains of the isolated domains. However, in real situations the individual domains will have been obtained from independent crystal structures or from homology models. Thus we have also tested pyDockTET over a docking sub-set of 20 cases, in which subunits were modelled from the structure of homologues (see Methods). As can be seen in Table 1, the pyDockTET predictions were always better (or in any case similar, but never significantly worse) than those of pyDock. Indeed, we found eight cases where the top solution is reasonably close (RMSD < 10Å) to the X-ray structure, so in about one out of three cases we can have a reasonable trust in the best solution that is picked up by pyDockTET.

**Table 1:** Domain-domain assembly with pyDockTET using homology models or X-ray structures of the interacting domains

linker length	domain-domain PDB^a^	docking from cryst.^b^		Docking from models^c^	
		
		pyDock	pyDockTET	pyDock	pyDockTET
2	1b8p_A_158_159	1 (1.3)	1 (6.8)	6 (4.5)	6 (4.5)

4	1ar4_A_84_87	68 (2.2)	39 (2.2)	15 (5.5)	2 (5.5)
	1aw7_A_93_96	1 (2.2)	1 (2.2)	1 (3.2)	1 (3.2)
	1ffu_F_176_179	1 (1.9)	1 (1.9)	98 (9.6)	121 (9.6)

5	1b06_A_93_97	11 (3.9)	1 (3.9)	3 (4.2)	2 (7.5)

6	1dlu_B_263_268	1 (1.8)	1 (1.8)	1 (5.1)	1 (9.2)

7	1ca1_-_251_257	301 (4.8)	10 (4.8)	415 (7.7)	22 (7.8)
	1e5m_A_251_257	1 (1.3)	1 (1.3)	1 (3.6)	1 (3.6)
	1gk8_C_147_153	-	-	-	-

8	1j3n_A_244_251	1 (1.9)	1 (5.6)	1 (2.0)	1 (5.2)

9	1ee0_A_234_242	4 (8.1)	2 (8.1)	1 (2.9)	1 (2.9)
	1nez_A_180_188	-	-	803 (9.1)	101 (9.1)
	1s9v_B_88_96	-	-	-	-

11	1etp_B_90_100	809 (7.4)	204 (7.4)	1303 (9.8)	437 (9.8)

12	1onq_A_182_193	-	-	-	-

13	1edh_B_100_112	-	-	-	-

14	1hnf_-_97_110	-	-	-	-
	1mb8_A_173_186	-	-	1 (7.2)	1 (7.2)

15	1jk8_B_88_102	-	-	-	-

16	1k2d_B_87_102	-	-	-	-

^a ^The name of the structure is shown with the format: *(PDB ID)_(chain name)_(the first residue of the linker)_(the last residue of the linker)*

^b ^The best ranking of any acceptable solution from docking the crystal structures. RMSD (Å) of the second domain is shown in brackets. '-' means that there is no acceptable solutions (≤ 10Å) generated by ZDOCK.

^c ^The best ranking of any acceptable solution from docking the modelled structures. RMSD (Å) of the second domain is shown in brackets. '-' means that there is no acceptable solutions (≤ 10Å) generated by ZDOCK.

**Figure 6:**
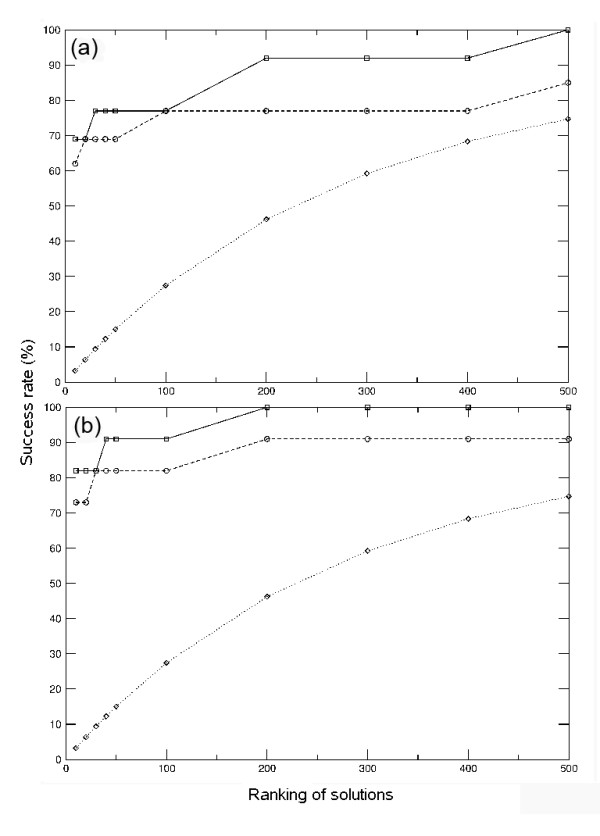
**The success rate of pyDock and pyDockTET for predicting crystal and modelled domain assemblies**. (a) The success rate of pyDock and pyDockTET in selecting at least one acceptable solution for a sub-set where domain structures have been modelled based on homologue templates, considering only those cases with at least one acceptable solution generated by ZDOCK. (b) The success rate of pyDock and pyDockTET in selecting at least one acceptable solution for the same sub-set when coordinates are taken from crystal structures, considering only those cases with at least one acceptable solution generated by ZDOCK. The solid lines with square markers are the results of pyDockTET, the dash lines with circle markers are the results of pyDock, the dotted lines with diamond markers are the results of random prediction.

In Figure 6 we compare the results on the sub-set of domains modelled from homologues, with the same sub-set of domains taken from the crystal structures. When we consider only the cases that have at least one acceptable solution generated by ZDOCK (13 out of 20), the success rates of pyDockTET for predicting an acceptable conformation in the top 10 and 50 solutions are 69% and 77%, respectively (success rates of pyDock alone are 62% and 69%, respectively) (Figure 6a). The top 10 and top 50 success rates for the same sub-set, when coordinates are taken from the crystal structure, are 82% and 91%, respectively (success rates of pyDock alone are 73% and 82%, respectively) (Figure 6b). In some of the cases we use templates with high sequence identity (see additional file 1: The 20 unbound (modelled) structures); however this does not significantly affect the results. Indeed, if we consider the cases that only have templates with maximum sequence identity of around 60% (1b8p, 1ar4, 1ffu, 1e5m, 1j3n, 1etp, and 1hnf), the pyDockTET success rates for the top 10 and 50 solutions are both 67% (as compared to 67% and 83%, respectively, for the same cases when using the crystal structures). In addition, if we also include the cases that have templates up to 80% sequence identity (1b06, 1nez, 1s9v, and 1jk8) the results do not significantly change either. Thus the overall results of domain docking do not seem to depend too much on the details of the models. Table 1 shows that most of the cases with poor pyDockTET predictions (1gk8, 1nez, 1s9v, 1etp, 1onq, 1edh, 1hnf, 1jk8, 1k2d) had similarly bad predictions when using the X-ray structures. There is only one case (1ffu) where results were far worse when using modelled domains than when using the X-ray structures, but these bad results did not seem to arise from low-quality modelling, since the modelled domains were 0.84Å and 0.65Å Cα RMSD with respect to the corresponding X-ray structures (see additional file 1: The 20 unbound (modelled) structures). The important issue is that, in most cases, the pyDockTET results we obtained when docking domain models were similarly good to those when docking X-ray structures. Moreover, there are even cases where modelled domains yielded better predictive rates than X-ray structures (1ar4, 1mb8). So in general, the domain models generated in an automatic way seem to be of sufficient quality to get similar docking results as when using the X-ray structures (this is likely because the X-ray structures of the unbound molecules differ anyway from those in the complexes).

### Comparison to other domain-domain assembly approaches

We have evaluated the performance of pyDockTET with respect to other computational methods that have been recently reported for domain-domain assembly. Lise et al. [7] tested their contact prediction method by generating 10 domain-domain orientations with the docking server GRAMM-X. They found an acceptable solution (fraction of native contacts > 0.1) in 12 out of 20 cases. For 5 of these 12 cases, the best model (in terms of fraction of native contacts) was ranked first by their contact scoring function. We can test pyDockTET in this benchmark. However, most of the cases in their benchmark have two linkers between the domains. Our method is focused onto two domains joined by a single linker (which in principle have more flexibility) and it is not directly applicable to domain-domain interactions with two linkers. Thus, we have applied our method to the only three cases of their benchmark where the domains are joined by a single linker. When we used the close configurations (with their side-chains remodelled by SCWRL) we found acceptable solutions (RMSD ≤ 10Å) for two cases, 13pk and 1tfb, which were ranked 1 and 678, respectively. When we used the open configurations (with their side-chains remodelled by SCWRL), we found only one case with acceptable solutions, 1jmc. Lise et al. [7] used the open configuration (but not remodelling of the side-chains) and found an acceptable solution (fraction of native contacts > 0.1) for only one case, 1tfb, which ranked 3. As a note of caution, the overall results of Lise et al. [7] strongly depended on the ability of GRAMM to generate acceptable docking poses in such small number of alternative poses. Another difference between their method and ours that makes difficult the comparison is that they used the criterion of fraction of native contacts above 0.1 to define the acceptable solutions, while we use here the RMSD (equivalent to the ligand RMSD as defined in CAPRI) below 10Å. Both criteria are used in CAPRI, in combination also with the interface RMSD, but no by separate. Ligand RMSD is arguably a more restrictive parameter than fraction of native contacts. For instance, from the last round 15 of CAPRI  we can observe a significant number of cases that, in spite of having fraction of native contacts above 0.1, are incorrect predictions by the global CAPRI criteria (average false positive rate of 9%). On the contrary, virtually all cases with ligand RMSD below 10 Å are correct predictions (average false positive rate of 0%), and there are even some solutions with ligand RMSD above 10 Å that are still acceptable (e.g. 2 cases in target T32, and 1 case in target T36).

Inbar et al. [8] recently described their combinatorial docking approach (CombDock) for multi-domain and multi-molecular assembly. However, they reported only three cases of domain-domain docking (the other reported cases were either docking of secondary structure elements within a single domain, or multi-molecular docking): 1a47, 1b23, and 1d0n. For all of them they found near-native assemblies within the top 10 solutions. However, our method is not directly applicable to these cases, since they have more than two domains (we could dock one domain onto the other two domains taken as a single rigid-body, but that would not be a realistic test for our method).

Finally, Wollacott et al. [5] recently reported a domain-domain assembly method based on conformational sampling of the inter-domain linker with their Rosetta program. Although they did not use computational docking, they provided an interesting test set to evaluate the performance of our approach. They divided their benchmark set according to their predictive results. For 38 out of 76 cases, they had a near-native decoy with RMSD < 2 Å within the top five models (global success rate 50%). We applied our method to 18 of these successful cases (the other ones were defined as single domains by SCOP, or the linkers were too long for our method), and found acceptable solutions for 15 of them. The overall results of pyDockTET were consistent with the ones we obtained in our data set of 77 cases: six cases with an acceptable solution ranked as top one (global success rate 33%; success rate normalized for only those cases with acceptable solution 40%); nine cases with an acceptable solution within the top 10 solutions (global success rate 50%, normalized success rate 60%); and 11 cases with an acceptable solution within the top 50 (global success rate 61%; normalized success rate 73%). For another 18 cases, they had near-native decoys (RMSD <2 Å) within the top 250 models, but they did not give exact ranking numbers, so a direct comparison with our method would be difficult. For the remaining 20 cases, in 13 of them their method did not produce any acceptable solution at all, and in 7 of them there was no acceptable solution within the top 250 models (Tables 2 and 3 in Ref. [5]), so we wanted to check whether our method would be able to improve their results. We applied our method for eight of these difficult cases (we selected only those ones with linker length ≤ 17; we also excluded 1qla and 1qov because they were defined as single domain by both SCOP and Pfam), and found acceptable solutions for five of them. We found one case with an acceptable solution ranked as top one (global success rate 13%; normalized success rate 20%); four cases with an acceptable solution within the top 10 (global success rate 50%; normalized success rate 80%); and five cases with an acceptable solution within the top 50 (global success rate 63%; normalized success rate 100%). These results are also consistent with the ones obtained in the set of good cases in Wollacott et al. [5] and with those in our data set of 77 cases. Thus, our method failed for some of the cases for which Wollacott et al. [5] had excellent results, but succeeded in some of the cases where Wollacott et al. [5] did not have good predictions. The global success rate of pyDockTET for the top 10 solutions (global success rate 50%; normalized success rate 60–80%) is comparable to the global success rate for the top 5 solutions in Wollacott et al. [5] (global success rate 50%; normalized success rate 60%), although we have to say in their favour that they used a stricter definition of the acceptable solution.

**Table 2:** Domain docking results on difficult cases for domain-domain assembly

PDB	pyDock^a^	pyDockTET^a^	templates (dom 1/dom 2)^d^
1cx4	237 (14.6)^b^	129 (14.6)^b^	1wgp_A(1–123)/2cgp_A(8–207)
1pii	3 (14.0)^b^	6 (15.9)^b^	1jcm_P(4–253)/1nsj_-(1–201)
1qcs	1 (7.0)	1 (7.0)	1cr5_B(23–103)/1qdn_A(91–183)
1nkr	37 (10.3)^c^	6 (10.3)^c^	1m4k_A(8–102)/2dli_A(109–200)
1a6q	41 (10.8)^b^	2 (10.8)^b^	2p8e_A(4–295)/1q1v_A(309–378)
1clc	1291 (7.2)	39 (7.2)	1rq5_A(208–299)/1ks8_A(1–426)

^a ^The best ranking of the near-native prediction by pyDock and pyDockTET. The values in brackets are the RMSD value of the predicted solution.

^b ^No solution with RMSD < 10Å in the docking set. We show the best rank of the lowest-RMSD solutions.

^c ^A solution is found with RMSD 9.2Å that is ranked 168 by pyDock, and ranked 20 by pyDockTET.

^d ^The information of templates for modelling domain 1 (left) and 2 (right). The format of the template information is *PDB id *_*chain *(*sequence range*).

**Figure 7:**
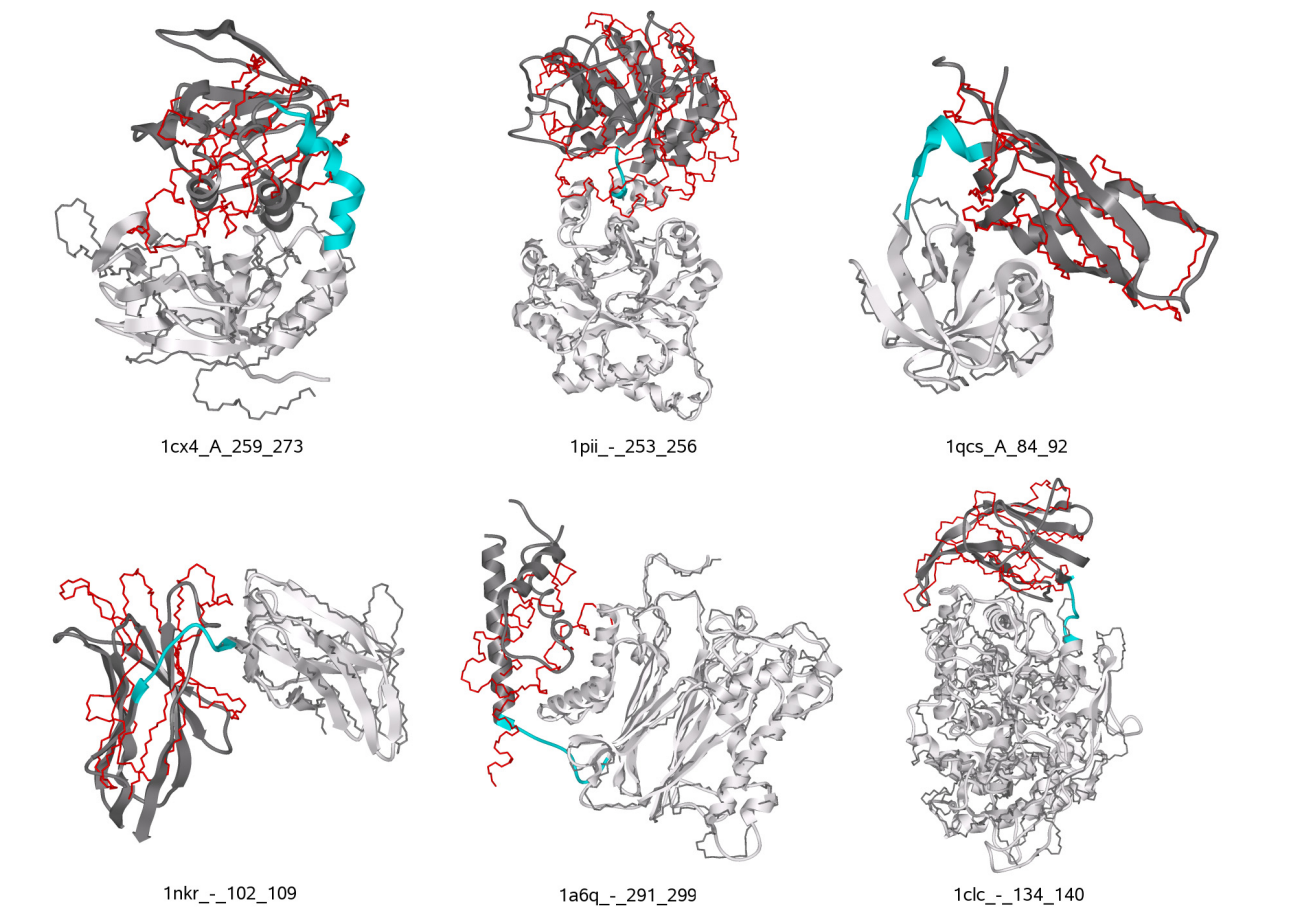
**Best models generated by pyDockTET for the difficult cases of Wollacott et al**.[5]. The modelled structure for each case is represented in stick mode, with the first domain in white colour. The second domain orientation predicted by pyDockTET is shown in red. The real X-ray structure is shown for comparison in ribbon mode: the first domain in white colour, the second domain in grey, and the linker in cyan. The first domains of the real and modelled structures are superimposed.

We also performed an additional test on the difficult cases of Wollacott et al. [5], by independently modelling the two domains based on different templates. Then we ran pyDockTET on these independently modelled domains, instead of on the X-ray structures. We excluded two additional cases (1crz and 1f5n) because they did not have any suitable homologous template according to our criteria (see Methods). Our final set was formed by the six cases shown in Table 2. In four of these six cases, we obtained a reasonable model within the top 10 docking solutions as ranked by pyDockTET (Table 2). We have to note that in most of the cases ZDOCK was not able to generate docking solutions with RMSD < 10Å, so we have considered as reasonable other orientations with larger RMSD with respect to the X-ray structure. Particularly interesting are cases 1qcs and 1a6q, where pyDockTET found acceptable docking solutions with rank 1 and 2, respectively. The best solutions for all these cases are shown in Figure 7. The success rates are quite encouraging, especially considering that these examples were highly challenging cases for other domain-domain assembly methods. Although we need to improve the sampling of docking orientations in order to better modelling these difficult cases, it is clear that the inclusion of distance restraints from linker lengths largely helps to model the assembly of multi-domain proteins.

## Conclusion

We have described here a procedure to build multi-domain proteins from the structure (experimental or modelled) of their individual domains, using a combination of rigid-body docking, binding energy scoring, and linker-length based distance restraints. The inclusion of linker-based distance restraints largely improves the structural predictions, especially for those cases where binding energy alone is not sufficient to discriminate the near-native conformations. Provided that the rigid-body generation method is able to produce acceptable domain-domain orientations, our scoring function (based on docking energy plus restraints) finds the correct assembly within the top 10 solutions in about 60–70 % of the cases.

## Methods

### Rigid-body docking and restraint-based scoring function

**Figure 8:**
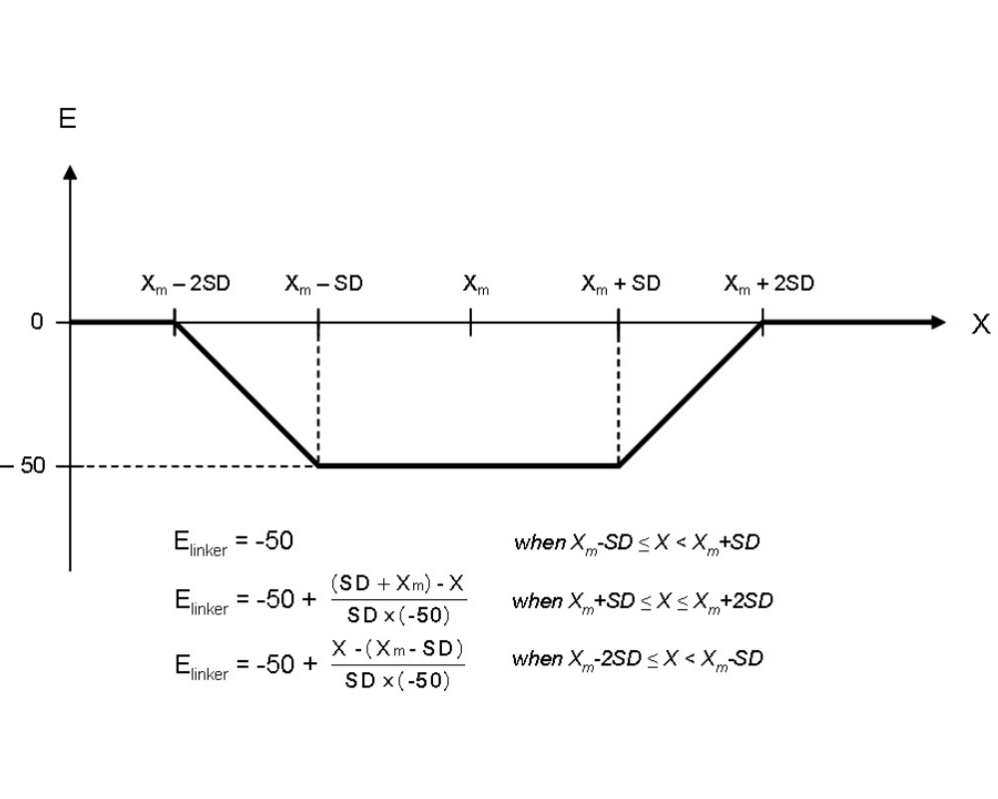
**The function of E_linker _of pyDockTET**. The function of E_linker _of pyDockTET, where X_m _is the average end-to-end distance of a linker with specific length, and SD is the standard deviation of the X_m_.

Rigid-body docking was performed on the interacting domains by ZDOCK2.1 [17]. The resulting domain-domain orientations were firstly evaluated with the standard pyDock protocol, which uses Coulombic electrostatics with distance-dependent dielectric constant plus ASA-based desolvation optimized for protein-protein docking as previously described [10]. Finally, the domain-domain docking poses are further scored with the module pyDockTET, which uses the average linker end-to-end distance, X_m_, as a restraint to select the correct docking poses. The X_m _value is calculated as the average of the end-to-end distance values of linkers that have same length (i.e. same number of residues) in the 542 linker structures collected from multi-domain proteins in Protein Data Bank (PDB). The X_m_ value and its corresponding standard deviation, SD, are then used to develop a function, E_linker _(Figure 8), which is further incorporated into the pyDock energy function for the final rescoring of domain-domain poses (equation 1).

(1) E = E_elec _+ E_desolv _+ E_linker _

where E_elec _represents electrostatics and E_desolv _represents desolvation energy.

### Predictive success rates evaluation

For a pair of domain structures we generated 2,000 rigid-body docking orientations by ZDOCK2.1 [17]. The scoring function was then tested by calculating the success rate of predicting a near-native solution among the N top rankings (N = 10, 20, 30, 40, 50, 100, 200, 300, 400, 500) as scored by pyDock (before restraints) and pyDockTET (after restraints). Following the criteria in CAPRI  for the assessment of results from protein-protein docking, here a near-native solution is considered acceptable if the RMSD of the one of the domains is ≤ 10Å from the equivalent one in the X-ray structure, when the other domain (typically the larger one) is superimposed onto that of the X-ray structure (similarly, a near-native solution is defined as a good one if the RMSD from the X-ray structure is ≤ 5Å).

### Domain-domain structural test set

A benchmark of 77 non-redundant domain pair structures was compiled by selecting all crystal structures of multi-domain proteins in PDB that satisfied the following criteria: i) since domain pairs that are not in direct contact cannot be predicted by our domain-domain docking, the benchmark cases were required to have at least one pair of residues that had side chain atoms within a distance ≤ 5Å in their crystal structures (see additional file 2: The 77 non-redundant bound structures); ii) all crystal structures had a resolution ≤ 2.5Å and less than 30% sequence identity to each other (this is a standard sequence identity threshold in homologue search); and iii) we considered only proteins formed by two domains as defined by Pfam, with a single inter-domain linker (the linker regions were thus defined by the domain boundaries of Pfam, and all the 77 non-redundant domain pairs contained linkers that covered the domain cutting sites defined by SCOP [13], or were near them within three amino acids difference). For a more realistic domain assembly test, we used SCWRL 3.0 [16] in order to re-model all side chains of the individual domains before docking.

The second benchmark set contained 20 non-redundant domain pairs in which each domain was modelled on the basis of a homologue. This sub-set was generated from the previously described benchmark of 77 pairs, after selecting those cases in which both domains had available templates and thus could be independently modelled. The modelling process applied BLAST [20] to search for template structures (considering only homologous sequences with the best E-values, as long as they are below the limit of 10^-20^) and used Baton (D. Burke, unpublished; based on the COMPARER algorithm [21]) to do multiple structural alignment of templates. Fugue [22] was used to find templates in those cases in which BLAST failed and also to generate all the sequence-structural alignments. Finally MODELLER [23] was used to generate models for each domain. The modelled cases are listed in Table 1. The template structures and the sequence identities (computed from the structural alignments) can be found in the additional file 1: The 20 unbound (modelled) structures.

## Authors' contributions

TLB and JFR devised the concept and directed the research. JFR designed the procedure. TMKC design the implementation of the program and performed the calculations. TMKC and JFR analysed the data. TMKC drafted the paper. TLB and JFR finalized the draft. All authors read and approved the final manuscript.

## Supplementary Material

Additional file 1 **The 20 unbound (modelled) structures**. Each structure is shown as (*PDB ID*)_(*chain*)_(*the first residue of the linker*)_(*the last residue of the linker*). Click here for file

Additional file 2 **The 77 non-redundant bound structures**. Each domain complex is shown as (*PDB ID*)_(*chain*)_(*the first residue of the linker*)_(*the last residue of the linker*). Click here for file
