# Supplementary material for: Structural assembly of two-domain proteins by rigid-body docking
Source: BMC Bioinformatics. 2008 Oct 16;9:441. doi: 10.1186/1471-2105-9-441 (PMC2579442; doi:10.1186/1471-2105-9-441)
Supplement: Additional file 1 — The 20 unbound (modelled) structures. Each structure is shown as (PDB ID)_(chain)_(the first residue of the linker)_(the last residue of the linker). [file 1471-2105-9-441-S1.doc]

Additional file 1: The 20 unbound (modelled) structures.

Each structure is shown as (*PDB ID*)_(*chain*)_(*the first residue of the linker*)_(t*he last residue of the linker*)

| Linker length | domain1/domain2 struture | Template PDB structure | Sequence identity *^a^* (%) |
| --- | --- | --- | --- |
| 2 | 1b8p_A_158_159_domain1 | 1bdm_A_1_332 | 54.1 |
|  |  | 1bmd_A_1_332 | 58.0 |
|  |  | 1civ_A_12_385 | 40.1 |
|  |  | 4mdh_A_1_333 | 49.7 |
|  |  | 5mdh_A_1_333 | 49.7 |
|  |  | 7mdh_A_23_385 | 38.9 |
|  | 1b8p_A_158_159_domain2 | 1bdm_A_1_332 | 45.9 |
|  |  | 1bmd_A_1_332 | 46.5 |
| 4 | 1ar4_A_84_87_domain1 | 1gn3_A_2_199 | 61.2 |
|  |  | 1gn4_A_2_199 | 61.2 |
|  |  | 1gn6_A_2_199 | 61.2 |
|  | 1ar4_A_84_87_domain2 | 1gn2_A_2_199 | 54.3 |
|  |  | 1gn3_A_2_199 | 54.3 |
|  |  | 1gn6_A_2_199 | 54.3 |
|  | 1aw7_A_93_96_domain1 | 1qil_A_1_194 | 88.3 |
|  |  | 1ts2_A_1_194 | 88.3 |
|  |  | 1ts4_A_1_194 | 88.3 |
|  |  | 1ts5_A_1_194 | 89.4 |
|  |  | 2qil_A_1_194 | 89.4 |
|  |  | 3tss_5_194 | 93.6 |
|  | 1aw7_A_93_96_domain2 | 1qil_A_1_194 | 87.0 |
|  |  | 1ts2_A_1_194 | 87.0 |
|  |  | 1ts4_A_1_194 | 88.0 |
|  |  | 1ts5_A_1_194 | 88.0 |
|  |  | 2qil_A_1_194 | 88.0 |
|  |  | 3tss_5_194 | 92.0 |
|  | 1ffu_F_176_179_domain1 | 1fiq_B_224_528 | 23.2 |
|  |  | 1n5w_C_1_287 | 52.5 |
|  | 1ffu_F_176_179_domain2 | 1fiq_B_224_528 | 13.6 |
|  |  | 1n5w_C_1_287 | 52.7 |
| 5 | 1b06_A_93_97_domain1 | 1wb7_A_4_208 | 75.0 |
|  |  | 1wb8_A_4_208 | 76.1 |
|  | 1b06_A_93_97_domain2 | 1p7g_A_12_222 | 45.2 |
|  |  | 1wb7_A_4_208 | 71.3 |
|  |  | 1wb8_A_4_208 | 71.3 |
| 6 | 1dlu_B_263_268_domain1 | 1m1o_A_3_392 | 78.5 |
|  |  | 1m1t_A_1_392 | 78.2 |
|  |  | 1m4s_A_1_392 | 78.5 |
|  |  | 1qfl_A_4_392 | 78.5 |
|  |  | 1wl4_A_4_397 | 50.2 |
|  |  | 2f2s_A_32_427 | 33.7 |
|  | 1dlu_B_263_268_domain2 | 1m1o_A_3_392 | 88.1 |
|  |  | 1m1t_A_1_392 | 88.1 |
|  |  | 1m4s_A_1_392 | 88.1 |
|  |  | 1qfl_A_4_392 | 88.1 |
| 7 | 1ca1_-_251_257_domain1 | 1kho_A_1_369 | 72.2 |
|  |  | 1olp_A_1_369 | 52.8 |
|  |  | 1qm6_A_1_369 | 85.3 |
|  | 1ca1_-_251_257_domain2 | 1kho_A_1_369 | 73.0 |
|  |  | 1olp_A_1_369 | 46.1 |
|  |  | 1qm6_A_1_369 | 82.6 |
|  | 1e5m_A_251_257_domain1 | 1kas_2_412 | 48.2 |
|  |  | 1ox0_A_-5_409 | 42.5 |
|  |  | 2alm_A_-2_409 | 42.5 |
|  | 1e5m_A_251_257_domain2 | 1kas_2_412 | 48.4 |
|  |  | 1w0i_A_31_461 | 44.1 |
|  |  | 2c9h_A_18_444 | 45.3 |
|  | 1gk8_C_147_153_domain1 | 1uw9_A_11_475 | 87.9 |
|  |  | 1uwa_A_11_475 | 87.9 |
|  | 1gk8_C_147_153_domain2 | 1uw9_A_11_475 | 89.7 |
|  |  | 1uwa_A_11_475 | 90.0 |
| 8 | 1j3n_A_244_251_domain1 | 1kas_2_412 | 47.3 |
|  |  | 1ox0_A_-5_409 | 41.2 |
|  |  | 2alm_A_-2_409 | 41.2 |
|  | 1j3n_A_244_251_domain2 | 1kas_2_412 | 61.6 |
|  |  | 1ox0_A_-5_409 | 47.8 |
|  |  | 2alm_A_-2_409 | 47.2 |
| 9 | 1ee0_A_234_242_domain1 | 1chw_A_1_389 | 47.9 |
|  |  | 1cml_A_1_389 | 47.9 |
|  |  | 1qlv_A_18_395 | 72.6 |
|  | 1ee0_A_234_242_domain2 | 1i88_A_1_389 | 57.4 |
|  |  | 1i89_A_2_389 | 58.1 |
|  |  | 1qlv_A_18_395 | 85.8 |
|  | 1nez_A_180_188_domain1 | 1k8d_A_1_274 | 54.1 |
|  |  | 1ld9_A_1_268 | 54.7 |
|  |  | 1ldp_H_1_272 | 54.7 |
|  | 1nez_A_180_188_domain2 | 1c16_A_1_276 | 77.3 |
|  |  | 1ddh_A_1_274 | 76.1 |
|  |  | 1qo3_A_2_275 | 77.3 |
|  | 1s9v_B_88_96_domain1 | 1jk8_B_3_192 | 75.9 |
|  |  | 1uvq_B_3_191 | 67.8 |
|  | 1s9v_B_88_96_domain2 | 1d9k_D_2_189 | 70.8 |
|  |  | 1jk8_B_3_192 | 78.7 |
|  |  | 1uvq_B_3_191 | 77.5 |
| 11 | 1etp_B_90_100_domain1 | 1cno_A_1_86 | 2.2 |
|  |  | 1fcd_C_1_174 | 23.1 |
|  |  | 2mta_C_1_147 | 5.5 |
|  | 1etp_B_90_100_domain2 | 1cno_A_1_86 | 2.2 |
|  |  | 1fcd_C_1_174 | 23.9 |
|  |  | 2mta_C_1_147 | 6.5 |
| 12 | 1onq_A_182_193_domain1 | 1gzp_A_4_279 | 42.9 |
|  |  | 1xz0_A_7_277 | 79.7 |
|  |  | 1zt4_A_6_277 | 34.5 |
|  | 1onq_A_182_193_domain2 | 1gzp_A_4_279 | 78.7 |
|  |  | 1xz0_A_7_277 | 86.5 |
|  |  | 1zt4_A_6_277 | 77.5 |
| 13 | 1edh_B_100_112_domain1 | 1ff5_A_-1_218 | 89.9 |
|  |  | 1q1p_A_2_213 | 89.9 |
|  | 1edh_B_100_112_domain2 | 1ff5_A_-1_218 | 90.3 |
|  |  | 1q1p_A_2_213 | 89.3 |
| 14 | 1mb8_A_173_186_domain1 | 1dxx_A_9_246 | 47.1 |
|  |  | 1sh5_A_6_237 | 75.6 |
|  |  | 1wku_A_42_266 | 42.9 |
|  | 1mb8_A_173_186_domain2 | 1sh5_A_2_6_237 | 86.2 |
|  |  | 1sh5_A_6_237 | 86.2 |
|  | 1hnf_-_97_110_domain1 | 1ccz_A_1_171 | 15.8 |
|  |  | 1l6z_A_1_203 | 17.9 |
|  |  | 2dru_A_1_180 | 17.9 |
|  |  | 2if7_A_3_193 | 18.9 |
|  | 1hnf_-_97_110_domain2 | 1ccz_A_1_171 | 55.4 |
|  |  | 2dru_A_1_180 | 52.7 |
| 15 | 1jk8_B_88_102_domain1 | 1lnu_B_1_217 | 65.5 |
|  |  | 1s9v_B_3_189 | 69.0 |
|  |  | 1uvq_B_3_191 | 77.0 |
|  | 1jk8_B_88_102_domain2 | 1k2d_B_5_189 | 75.0 |
|  |  | 1s9v_B_3_189 | 79.3 |
|  |  | 1uvq_B_3_191 | 76.1 |
| 16 | 1k2d_B_87_102_domain1 | 1d9k_D_2_189 | 78.0 |
|  |  | 1f3j_B_4_191 | 76.8 |
|  |  | 1iak_B_5_189 | 75.6 |
|  | 1k2d_B_87_102_domain2 | 1d9k_D_2_189 | 83.3 |
|  |  | 1iak_B_5_189 | 84.4 |
|  |  | 1lnu_B_1_217 | 82.2 |

^a^ Sequence identity is computed for the model in the context of the multiple structural alignment of the templates.
