# Supplementary material for: Structural assembly of two-domain proteins by rigid-body docking
Source: BMC Bioinformatics. 2008 Oct 16;9:441. doi: 10.1186/1471-2105-9-441 (PMC2579442; doi:10.1186/1471-2105-9-441)
Supplement: Additional file 2 — The 77 non-redundant bound structures. Each domain complex is shown as (PDB ID)_(chain)_(the first residue of the linker)_(the last residue of the linker). [file 1471-2105-9-441-S2.doc]

Additional file 2: The 77 non-redundant bound structures.

Each domain complex is shown as (*PDB ID*)_(*chain*)_(*the first residue of the linker*)_(t*he last residue of the linker*)

| 1a8p_-_102_106 | 1ah5_-_218_224 | 1amm_-_82_88 | 1aor_B_208_216 | 1aqh_-_350_358 | 1ar4_A_84_87 |
| --- | --- | --- | --- | --- | --- |
| 1aw7_A_93_96 | 1aw9_-_78_93 | 1b06_A_93_97 | 1b25_A_206_214 | 1b4a_A_71_78 | 1b8p_A_158_159 |
| 1b9k_A_820_824 | 1bag_-_343_358 | 1bay_A_75_83 | 1bht_B_425_427 | 1bik_-_78_80 | 1ca1_-_251_257 |
| 1cfb_-_701_712 | 1chm_B_157_164 | 1clc_-_131_137 | 1clv_A_375_382 | 1cr5_B_106_119 | 1dlu_B_263_268 |
| 1e5m_A_251_257 | 1e9i_B_134_141 | 1ebg_A_134_142 | 1edh_B_100_112 | 1ee0_A_234_242 | 1epf_A_97_112 |
| 1et6_B_96_100 | 1et9_A_95_99 | 1etp_B_90_100 | 1f2e_A_76_88 | 1fdr_-_93_105 | 1ffh_-_88_96 |
| 1ffu_F_176_179 | 1fiq_B_413_417 | 1fpo_A_75_85 | 1ghq_B_64_71 | 1gk8_C_147_153 | 1grj_-_75_79 |
| 1gsq_-_73_81 | 1h03_Q_65_68 | 1h1o_B_288_300 | 1hdm_B_83_88 | 1hnf_-_97_110 | 1i8d_B_88_99 |
| 1ik6_A_185_197 | 1j3n_A_244_251 | 1j5y_A_68_74 | 1jk8_B_88_102 | 1jlv_A_75_87 | 1k2d_B_87_102 |
| 1kgz_A_78_82 | 1kzl_A_88_99 | 1lsl_A_472_477 | 1mb8_A_173_186 | 1n5w_F_176_181 | 1nez_A_180_188 |
| 1oe7_A_81_89 | 1oi7_A_115_131 | 1onq_A_182_193 | 1p5u_A_149_151 | 1pbj_A_58_68 | 1pdy_-_134_140 |
| 1pgs_-_141_148 | 1pii_-_253_256 | 1pmt_-_76_86 | 1qg3_A_1209_1219 | 1qnn_A_83_85 | 1r5a_A_77_89 |
| 1r6l_A_145_157 | 1rwz_A_115_120 | 1s9v_B_88_96 | 1v8g_A_65_69 | 1vk6_A_125_126 |  |
